# Hyperventilation-induced heart rate response as a potential marker for cardiovascular disease

**DOI:** 10.1038/s41598-019-54375-9

**Published:** 2019-11-29

**Authors:** Selwynne M. Hawkins, Dominik P. Guensch, Matthias G. Friedrich, Giulia Vinco, Gobinath Nadeshalingham, Michel White, Francois-Pierre Mongeon, Elizabeth Hillier, Tiago Teixeira, Jacqueline A. Flewitt, Balthasar Eberle, Kady Fischer

**Affiliations:** 1 Research Institute of the McGill University Health Centre, Montreal, Canada; 2 Department of Anaesthesiology and Pain Medicine, Bern University Hospital, Inselspital, University of Bern, Bern, Switzerland; 3 Department of Diagnostic, Interventional and Paediatric Radiology, Bern University Hospital, Inselspital, University of Bern, Bern, Switzerland; 4 Departments of Medicine and Diagnostic Radiology, McGill University Health Centre, Montreal, Canada; 5 Department of Family Medicine, McGill University, Montreal, Canada; 6 Departments of Cardiac Sciences and Radiology, University of Calgary, Calgary, Canada; 7 University of Verona, Verona, Italy; 8 Philippa and Marvin Carsley CMR Center at the Montreal Heart Institute, Montreal, Canada; 9 Stephenson Cardiovascular MR Centre, Libin Cardiovascular Institute of Alberta, Calgary, Canada; 10 Douro e Vouga Hospital Centre, Sta Maria da Feira, Portugal

**Keywords:** Heart failure, Diagnostic markers

## Abstract

An increase of heart rate to physical or mental stress reflects the ability of the autonomous nervous system and the heart to respond adequately. Hyperventilation is a user-controlled breathing maneuver that has a significant impact on coronary function and hemodynamics. Thus, we aimed to investigate if the heart rate response to hyperventilation (HRR_HV_) can provide clinically useful information. A pooled analysis of the HRR_HV_ after 60 s of hyperventilation was conducted in 282 participants including healthy controls; patients with heart failure (HF); coronary artery disease (CAD); a combination of both; or patients suspected of CAD but with a normal angiogram. Hyperventilation significantly increased heart rate in all groups, although healthy controls aged 55 years and older (15 ± 9 bpm) had a larger HRR_HV_ than each of the disease groups (HF: 6 ± 6, CAD: 8 ± 8, CAD+/HF+: 6 ± 4, and CAD−/HF−: 8 ± 6 bpm, p < 0.001). No significant differences were found between disease groups. The HRR_HV_ may serve as an easily measurable additional marker of cardiovascular health. Future studies should test its diagnostic potential as a simple, inexpensive pre-screening test to improve patient selection for other diagnostic exams.

## Introduction

Cardiovascular disease affects nearly one third of people over the age of 65^1^ in developed countries and places considerable financial strain on health care systems^2,3^. As a result, research focused on predictive metrics of cardiovascular disease is paramount^4^. Presently, several diagnostic tests are used—including stress tests and coronary angiograms—to verify or exclude the presence of cardiovascular disease. However, these approaches have many limitations, including higher costs, intricate diagnostic equipment, exposure to radiation, invasive procedures, or injections of pharmacological agents. These factors restrict their clinical utility. Tests like 12-lead ECG and physical exams are more easily accessible, but often provide less information than imaging modalities^5^. The addition of a simple pre-screening test, performed without supplemental imaging techniques, could supplement the information gained through existing screening tests and reduce the number of costly advanced diagnostic tests.

Heart rate (HR), an accessible, quantifiable measure, has long been investigated as a predictor of cardiovascular disease and mortality^6,7^. A higher resting HR has been shown to be correlated with higher risk of cardiovascular disease and death in the general population and several patient groups, including heart failure with preserved ejection fraction (HFpEF)^8^ and coronary heart disease^9,10^. Another similar assessment associated with cardiovascular disease is HR variability, measured through stationary or ambulatory recordings^11–13^. Such parameters however may not consistently be abnormal at rest. Therefore, inducible HR changes as a response to physiological stress could provide useful incremental information. The response of the heart rate to stress, commonly exercise or pharmacological injection, has been shown to hold prognostic and diagnostic power. An inadequate heart rate response to increased activity, chronotropic incompetence, is especially prevalent in patients with heart failure (HF)^14^. The heart rate response to exercise has been shown to distinguish HF patients from age-matched controls and those with hypertension^15^, while a large outcome based analysis of the general population demonstrated that the magnitude of the heart rate response increase following exercise was a predictor of sudden cardiac death^16^. The heart rate response to adenosine has also been associated with advanced myocardial imaging results as one of the strongest negative predictors of reduced coronary flow reserve in women with non-obstructive coronary artery disease^17^. Additionally, a diminished heart rate response to adenosine infusion was an incremental prognostic marker for cardiovascular outcomes beyond traditional clinical and imaging risk factors, especially for women^18^. A heart rate response can also be induced through voluntary hyperventilation without the need for exercise of an injected agent. In nineteen controls we previously reported that while adenosine increased HR by 18 ± 14 bpm, an increase of 25 ± 14 bpm was also observed with hyperventilation^19^, although it is so far unknown if this is related to cardiovascular health.

Recently, cardiovascular magnetic resonance (CMR) studies by our groups and collaborators have used hyperventilation as a tool to investigate myocardial oxygenation and heart disease^19–21^. CMR, while considered the gold standard modality for many cardiac function and tissue parameters, is not suitable as an initial diagnostic or screening test because of the associated cost. On the other hand, the response of the heart rate to hyperventilation (HRR_HV_) would not require any equipment other than a device to measure heart rate. To investigate whether the HRR_HV_ may have potential as a preliminary indicator of cardiovascular health, we undertook a retrospective pooled analysis to investigate if differences in heart rate responses differ between controls and patient groups with CAD and HF.

## Results

### Study population

The baseline characteristics of healthy subjects and patients are summarized in Table 1. In total, 161 healthy controls, of which 30 participants were aged 55 years and older, and 121 patients were included. Of the patients, n = 54 were classified as CAD + , n = 34 as HF + , with n = 18 as having both CAD and HF (CAD + /HF + ). The remaining n = 15 were classified into the CAD−/HF− group as patients who presented with CAD symptoms but had no heart failure symptoms or significant coronary stenosis as evaluated via angiography. Large proportions of our patients were taking beta blockers, calcium channel antagonists, angiotensin converting enzyme (ACE) inhibitors, angiotensin-2 receptor blockers (ARB), and statins. In two studies, participants however were instructed to refrain from taking beta blockers on the morning of their CMR scan; in all other studies, participants continued medication normally, as defined by local guidelines.

**Table 1:** Participant Demographics

	All Controls (n = 161)	Controls 55 + (n = 30)	CAD (n = 54)	HF (n = 34)	CAD+/ HF + (n = 18)	CAD−/HF− (n = 15)
**Participant Characteristics**						
Age (years)	41.0 ± 13.9	59.8 ± 4.6	63.0 ± 10.2	61.5 ± 10.8	63.4 ± 9.1	61.6 ± 9.3
Gender (female)	71 (44%)	11 (37%)	6 (11%)*	15 (45%)	1 (6%)	5 (33%)
BMI (kg/m^2^)	24.7 ± 3.2	25.9 ± 3.0	27.9 ± 4.3	30.3 ± 6.2*	28.0 ± 3.6	26.7 ± 3.7
Systolic blood pressure (mmHg)	124.8 ± 14.9	132.3 ± 15.2	135.4 ± 15.8	124.2 ± 20.0	126.1 ± 20.2	127.7 ± 18.2
Diastolic blood pressure (mmHg)	77.4 ± 11.3	84.3 ± 12.5	78.7 ± 12.3	74.8 ± 9.5*	75.9 ± 11.1*	72.2 ± 9.0*
Resting heart rate (bpm)	62.7 ± 8.6	60.8 ± 7.5	64.4 ± 11.1	64.3 ± 11.1	62.1 ± 11.9	59.2 ± 6.8
Ejection fraction (%)	63.6 ± 6.3	64.8 ± 6.3	63.1 ± 8.5^†^	53.3 ± 13.3*	54.9 ± 11.8*	69.0 ± 8.6
Cardiac index (L/min/m^2^)	3.1 ± 0.7	2.9 ± 0.7	3.0 ± 0.9^†^	2.6 ± 0.6	2.8 ± 1.1	2.7 ± 0.4
**Comorbidities**						
Diabetes	—	—	15 (28%)	9 (27%)	8 (44%)	0 (0%)
Smoking	—	—	6 (11%)	6 (18%)	5 (28%)	1 (7%)
History of coronary artery reperfusion	—	—	15 (28%)	0 (0%)	15 (83%)	0 (0%)
Sleep apnea	—	—	5 (9%)	2 (6%)	2 (11%)	2 (13%)
Dyslipidemia	—	—	33 (61%)	14 (42%)	11 (61%)	10 (71%)
**Medication**						
Calcium channel antagonists	—	—	12 (22%)	3 (9%)	1 (6%)	0 (0%)
ACE inhibitors	—	—	13 (24%)	8 (24%)	6 (33%)	0 (0%)
Statins	—	—	42 (78%)	13 (39%)	15 (83%)	10 (67%)
Beta blockers	—	—	36 (66%)	22 (66%)	13 (72%)	8 (53%)
Taken on exam day	—	—	12 (22%)	14 (42%)	12 (67%)	0 (0%)
Halted on exam day	—	—	24 (44%)	8 (24%)	1 (5%)	8 (53%)

Continuous variables are reported as mean ± SD. Categorical variables are reported as the number of occurrences in each group (n(%)). Furthermore, data for healthy controls are shown for the entire group, as well as for the sub-group of controls aged 55 years and older. *p < 0.05 indicates a significant differences between controls aged ≥ 55 years and each diseased group. ^†^Ejection fraction and cardiac index were only available for 16/54 CAD patients. ACE: angiotensin-converting enzyme, BMI: body mass index.

### Hyperventilation response

**Figure 1:**
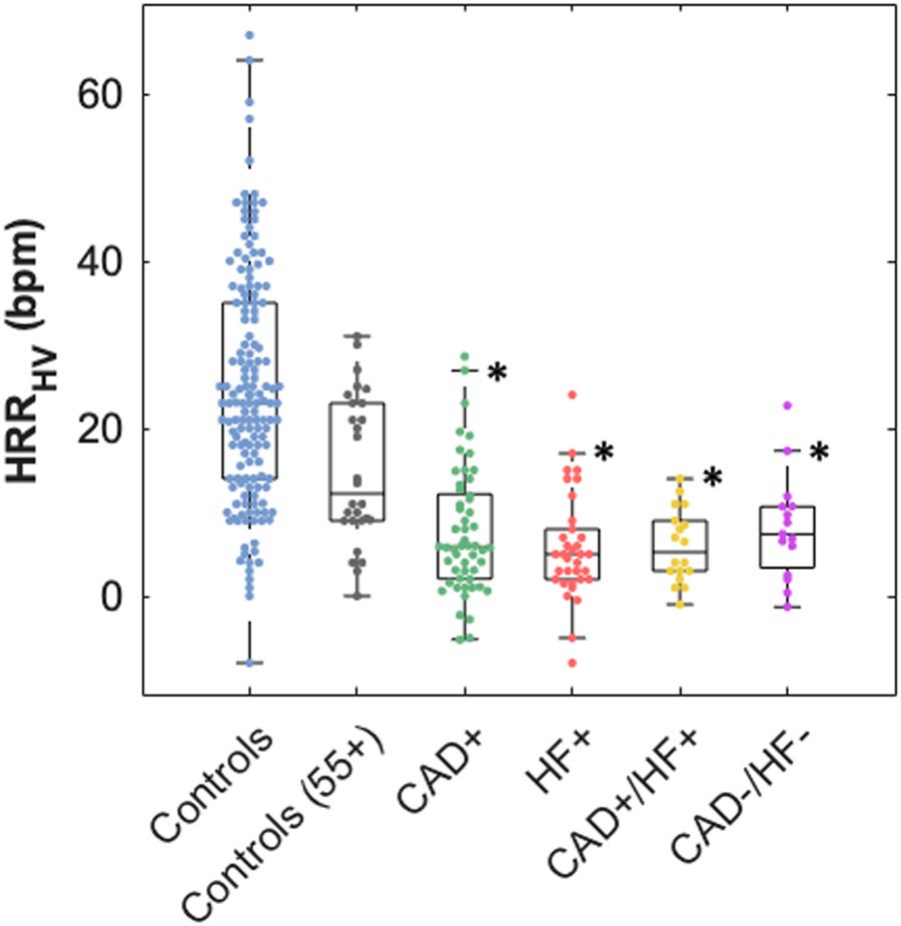
Heart Rate Response to Hyperventilation. Group medians with the interquartile range are depicted. All cardiovascular patient groups had a significantly attenuated HRR_HV_ in comparison to the response of the control sub-group 55 years and older (*p < 0.05).

All groups had a significant increase in heart rate between rest and post-hyperventilation (Table 2). The entire healthy control group composed of participants of all ages had an HRR_HV_ of 25 ± 14 bpm, while the ≥55 years control sub-group yielded an HRR_HV_ of 15 ± 9, and this was significantly greater than any HRR_HV_ observed in the patient groups (Fig. 1). Between the patient groups, no significant differences were found. When combining all patients (n = 121), the HRR_HV_ was 7 ± 7 bpm.

**Table 2:** Baseline Heart Rate and Heart Rate Response to Hyperventilation (HRR_HV_).

	All Controls	Controls 55 +	CAD	HF	CAD+/HF+	CAD−/HF−
Resting HR (bpm)	62.7 ± 8.6	61.2 ± 7.2	64.4 ± 11.1	64.3 ± 10.9	62.1 ± 11.9	59.2 ± 6.8
Post-Hyperventilation HR (bpm)	87.4 ± 15.8	76.2 ± 10.4	72.1 ± 12.1	70.0 ± 11.3	68.1 ± 10.8	67.3 ± 8.0
HRR_HV_ (bpm)	24.5 ± 13.8	15.0 ± 8.8	7.6 ± 7.5	5.7 ± 6.4	6.0 ± 4.4	8.2 ± 6.3
p-value	<0.001	<0.001	<0.001	<0.001	<0.001	<0.001

Mean ± SD resting heart rate (HR), post-hyperventilation HR and heart rate response (HRR_HV_). A paired t-test between resting and post-hyperventilation HR indicated a significant difference between baseline and post hyperventilation in each group.

### Impact of possible confounders

**Figure 2:**
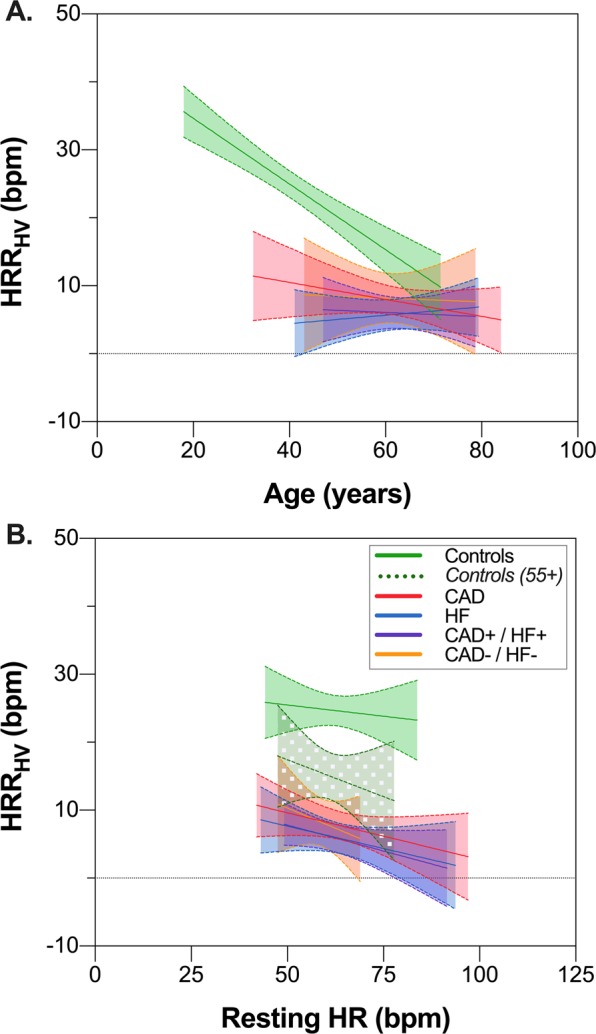
Impact of Age and Resting HR on HRR_HV_. (**A**) Heart Rate Response to Hyperventilation **(**HRR_HV_) as a function of age for healthy controls and each of the patient groups. (**B**) HRR_HV_ as a function of resting HR for healthy controls (Solid green: all controls, dashed green: sub-group of controls 55 years and older) and each of the disease groups. Solid lines indicate linear fit, and dashed lines indicate 95% confidence bounds on the fit.

**Figure 3:**
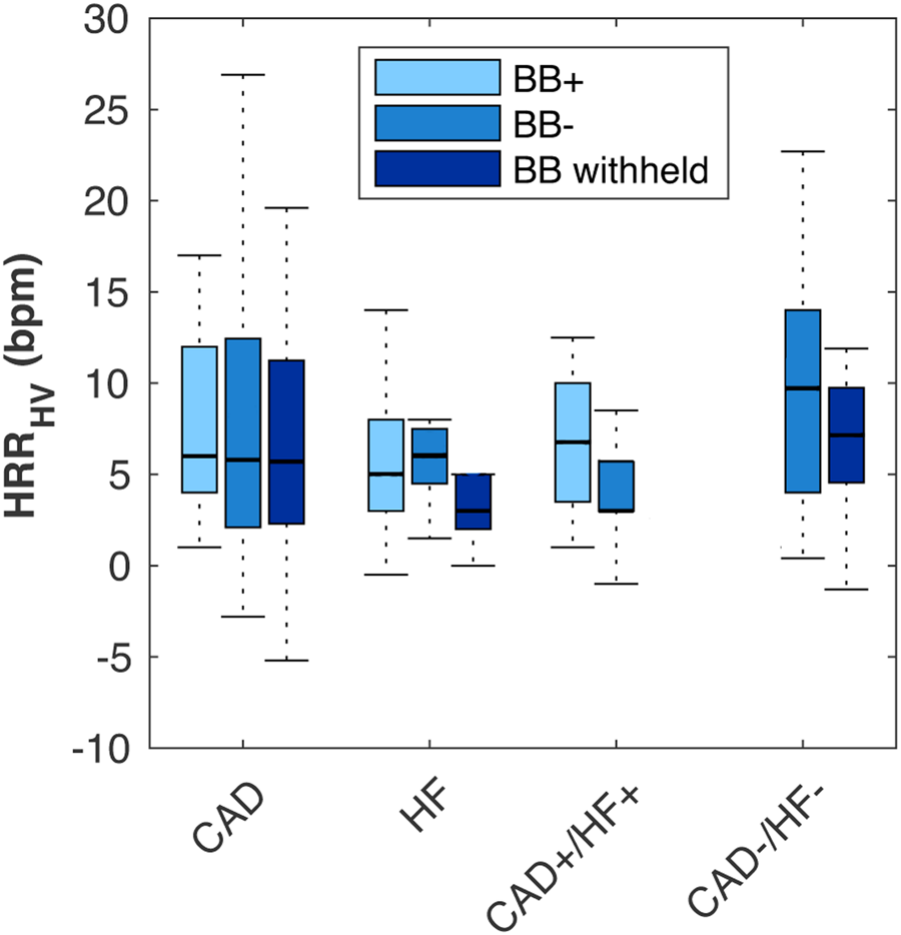
Impact of Beta Blocker Intake on the HRR_HV_. Box plot for the hyperventilation-induced heart rate response (HRR_HV_) in each group, where beta blocker status is indicated by colour. BB + indicates that beta blockers were taken the day of the study, while BB- indicates people who were not prescribed beta blockers at the time of the study. Sample size for each is indicated in Table 1.

Age had an inverse relationship with heart rate change in healthy controls, but not in patients with cardiovascular disease. As shown in Fig. 2A, the difference between groups was smaller with increasing age. Similarly, cardiac index had a positive relationship with HRR_HV_ in healthy controls of all ages, but not in the sub-group aged 55 years and older (Supplemental Fig. 1), while resting HR was not a significant factor for any group (Fig. 2B). Additionally, diabetes, obstructive sleep apnea and dyslipidemia were not associated with HRR_HV_ in the individual patient groups (Supplemental Fig. 1). The majority of the cardiovascular patient populations were taking beta blockers when recruited for their respective study (Table 1). A separation of the participants into three groups according to beta blocker status did not show a significant difference between participants taking beta blockers and those who did not (p = 0.647, Fig. 3).

### Reproducibility

**Figure 4:**
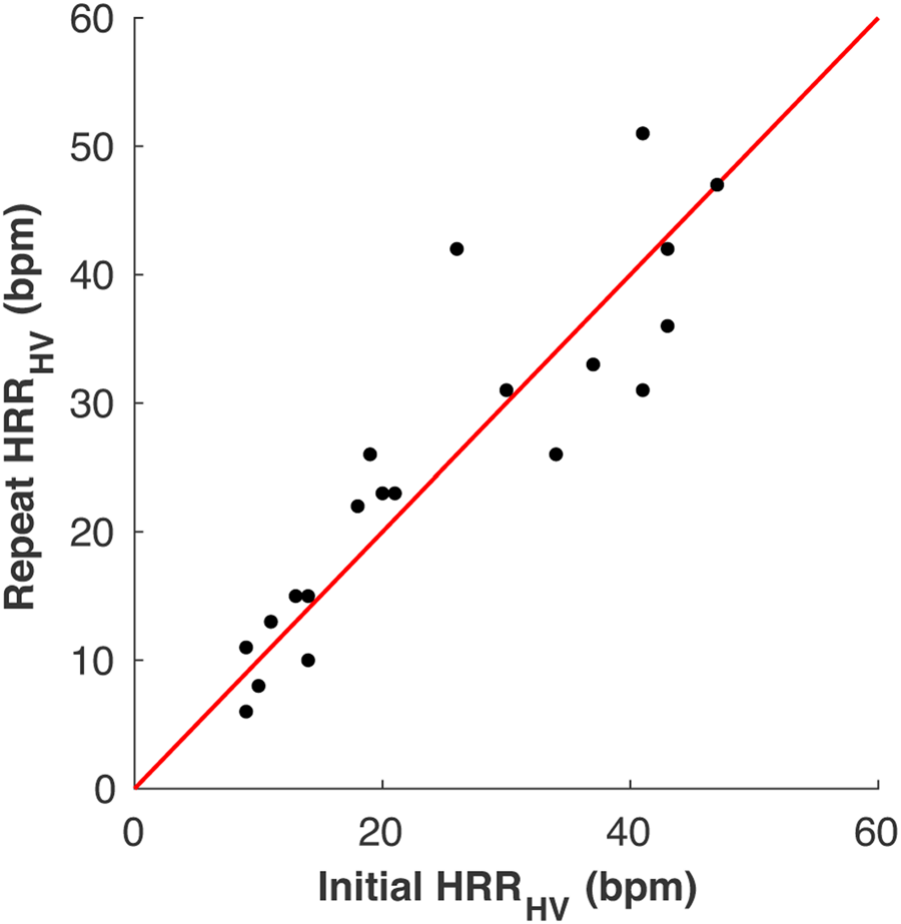
HRR_HV_ Reproducibility. Initial and secondary measures of hyperventilation-induced heart rate (HRR_HV_) changes in 20 healthy controls. ICC = 0.900, p < 0.001. The red line indicates the line of identity (x = y).

Reproducibility of HRR_HV_ was evaluated with data from 20 healthy controls who repeated the breathing maneuver twice in accordance with the protocol from their primary study (Fig. 4). The absolute intraclass correlation coefficient (ICC) was calculated to be 0.900 (p < 0.001), indicating good reliability, and the Lin concordance coefficient was calculated as 0.987, indicating good reproducibility.

### Preliminary diagnostic cut-offs

As no difference in HRR_HV_ was observed between patient groups, all cardiovascular patients were combined into a single group for a preliminary assessment of potential cut-offs (Supplemental Fig. 2). From these combined groups n = 54 controls and n = 54 patients were pseudo-randomly selected during the age-stratification process for inclusion into the ROC analysis (average age for controls: 54 ± 8; average age for combined patient group: 55 ± 8). With the age-stratified control group, a HRR_HV_ cut-off of 8.5 bpm resulted in a positive predictive value of 89 (78–95)% and a negative predictive value of 91 (80–96)% with an HRR_HV_ of 15.5 bpm for distinguishing from cardiovascular patients (Table 3).

**Table 3:** Potential HRRHV Diagnostic Cut-offs.

*HRR*_*HV*_	All Controls		Age-Stratified	
	8.5	15.5	8.5	15.5
Sensitivity	78% (72–84)	91% (85–95)	75% (63–84)	87% (72–94)
Specificity	87% (79–92)	72% (65–79)	86% (73–94)	69% (58–79)
Positive Predictive Value	92% (87–96)	73% (65–79)	89% (78–95)	59% (46–71)
Negative Predictive Value	67% (58–75)	91% (84–95)	70% (58–80)	91% (80–96)

The diagnostic potential of a lower hyperventilation-induced heart rate (HRR_HV_) cut-off of 8.5 bpm, and an upper cut-off of 15.5 bpm are shown with 95% confidence intervals for the comparison of all cardiovascular patients versus all controls and secondly for the age-stratified patients and control comparison.

## Discussion

Our pooled analysis of 9 studies revealed that 60 seconds of hyperventilation consistently induced a marked increase of heart rate in healthy controls and in patients with cardiovascular disease. However, the heart rate response to hyperventilation (HRR_HV_) was significantly diminished in cardiovascular patients (HRR_HV_ = 7 ± 7 for all patients), when compared to controls older than 55years (HRR_HV_ = 15 ± 9). No differences in HRR_HV_ were observed between cardiac disease groups.

Although not fully understood, these hyperventilation-induced HR changes are influenced by the interplay between several physiological control systems, specifically the autonomic nervous system. CAD patients have been observed to have impaired vagal function^12^, likely contributing to the attenuated HRR_HV_ seen in the CAD group. In our study, an attenuation of HRR_HV_ was also observed in HF patients. In HF, the underlying pathophysiology is strongly associated with decreased parasympathetic and increased sympathetic activity^11,22^, which could be contributing to the blunted heart rate response seen in the HF population. Decreased autonomic function, as seen in cardiovascular disease, also occurs with age^23^. Interestingly, the same attenuated HRR_HV_ was observed in the CAD−/HF− group, patients who presented with cardiac symptoms but no signs of heart failure or angiographically validated obstructive stenoses. However, despite the absence of HF or obstructive CAD, these are not healthy patients, as they can have an elevated risk of major adverse cardiac events and lower quality of life, especially if stress tests are abnormal. As shown in our analysis, HRR_HV_ in this CAD−/HF− group is in line with the classical heart disease groups of CAD and HF, and not healthy controls.

Resting HR has been shown to hold its own prognostic value for cardiovascular patients^24,25^. A higher resting HR could act as a confounder to the HRR_HV_. Specifically, HF patients often have a higher resting HR as a compensatory measure to counteract a decreased stroke volume in order to maintain cardiac output. In our study population, there was not a significant difference in resting heart rate between healthy controls and any patient group. This non-relationship was also observed in a large meta-analysis of controls for multiple methods of stress testing, including pharmacological stimuli and dynamic and isometric exercise^26^. Borlaug *et al*. similarly showed that patients with HFpEF did not have a different resting HR than age-matched hypertensive patients and controls, yet there was a significant difference in the exercise induced heart rate response, even with adjustment for beta blocker use^15^. Furthermore, a separate study showed the severity of HF was associated with a worsening exercise-induced heart rate response but neither the severity of HF or the heart rate response were associated with the resting HR^27^. In that study, both, resting HR and the heart rate response were associated with poor outcome despite a lack of a relationship between each other. In our analysis, the resting HR was not correlated with the hyperventilation-induced HR response in any subgroup. As there are small sample sizes for the individual pathologies in our study, this relationship should be further assessed with larger groups.

In the present study, with increasing age, there is a reduction in the HRR_HV_ in healthy controls which can make it more difficult to distinguish between groups (Fig. 2). In healthy controls, HRR_HV_ was diminished with age. A negative correlation of the stress induced heart rate reserve to age is well established^14,26–28^. This is in part due to the fact that maximal heart rate decreases approximately 4–5% per decade^29^, thereby reducing the heart rate response. Nevertheless, we still observed a significant attenuation in the patients in comparison to age-matched controls. While not the primary goal of the study, we also investigated potential diagnostic cut-off values. When using HRR_HV_ to separate age-stratified controls from the combined patient group, a lower HRR_HV_ cut-off of 8.5 yielded a positive predictive value of 89%, while an upper cut-off of 15.5 bpm resulted in a negative predictive value of 91%. These proposed cut-off values are preliminary and should be validated with a larger cohort of which the prevalence of cardiovascular disease is not pre-defined as it was in our analysis with specific sample sizes per group to truly test the role of HRR_HV_ as a discriminatory marker.

Furthermore, gender may play a role in heart rate responses to stimuli. It has been demonstrated that with pharmacological agents, women had a different heart rate response than men^17^. While Alexopolous *et al*. reported that with a prolonged 5-minute period of hyperventilation the heart rate response did not differ between male and female healthy controls, but multivariable analysis revealed gender was an independent predictor^30^. As shown in Supplemental Fig. 1, gender was not associated with the heart rate response to hyperventilation for any group, however we did not have the sample size to rigorously investigate this variable. Furthermore, there are known gender biases associated with some cardiovascular diseases that may influence comparisons between groups. For example, studies with CAD populations are often predominantly male^31^, while females have a higher or at least equal representation in studies of HFpEF^32^. These gender proportions are also reflected in our data. Other confounders are present as many common cardiovascular medications can impact the heart rate and its response to stress^33^, in particular beta blockers and non-dihydropyridine calcium channel blockers. Beta blockers did not have a significant effect on the HR response, regardless of whether they were taken, withheld, or not prescribed at all. In the literature, results on the impact of beta blockers on heart rate measurements are mixed. For example, the resting HR and HRR_HV_ in advanced HF was reported higher in patients when beta-blockers were withheld during treadmill exercise than when the test was repeated in the same patients 3 hours after beta blocker administration^34^, while other studies have reported that in HF patients, the heart rate response to stress was not associated with beta blocker use^27^. The effects of withholding beta blockers before stress testing has previously been examined in myocardial imaging, and it was shown that beta blocker withdrawal did not affect the diagnostic sensitivity^35^. Yet, there is little knowledge about the relationship with beta blockers and hyperventilation. In a larger population this impact will have to be assessed further, and different thresholds for patients on beta blockers may need to be developed, as this study is limited by power.

In patients with cardiovascular disease, several comorbidities occur with higher prevalence than in a healthy population. Frequent comorbidities such as diabetes, obesity, smoking and other cardiovascular risk factors are associated with impaired autonomic regulation. Hypertension is correlated with a higher resting HR^36^, and HR variability could hold prognostic power for the development of hypertension^36^. In diabetic patients, parasympathetic denervation is followed by reduced HR variability, resting tachycardia, and a fixed HR^37^. Obesity is correlated with reduced parasympathetic function and a higher resting HR. Similarly, smoking has been shown to be associated with a higher resting HR and a slower reactivity to exercise stimulus^38^. Cardiovascular disease is also closely linked with sleep apnea, with over half of clinically diagnosed sleep apnea patients also having a history of cardiovascular disease^39^. There is also a higher incidence of sleep apnea in HF and CAD patients as compared to healthy controls, with potential physiological links through autonomic dysfunction and impaired mechanical function^40^. Additionally, Cheyne-Stokes breathing is common in HF patients^41^, and recurrent hypercapnia and cardiopulmonary interaction can be associated with a highly periodic HR variation^42^. Each of these comorbidities affects the autonomic nervous system, potentially contributing to increased variability in hyperventilation-induced heart rate changes in the cardiovascular patient populations. Thus, it may be difficult to discern the etiology of hyperventilation-induced HR change between groups as it may reflect the impact of such comorbidities rather than cardiovascular disease itself.

In addition to the aforementioned limitations, the categorical classification of our patient groups as CAD, HF, CAD+/HF+, CAD−/HF−, or healthy precluded a statistical assessment of disease severity including the relationship with symptoms. For CAD patients only binary classification for presence of CAD was consistently available from all studies, preventing us from conducting a more complex subgroup analysis according to the severity, number and type of stenosed vessels. The limited sample size also required grouping of a variety of different disease profiles into the CAD+/HF + group, including those with any combination of current and re-perfused CAD. We also present a simple pooled analysis using data only from specified participating sites, and not a meta-analysis. The data is not weighted based on the origin of the study, and subsequently our analysis does not account for potential bias between studies and sites. Another limitation of this retrospective analysis was each study performed their own technique independently. While each site performed a 60 s period of hyperventilation, the standardization of the protocol between sites was not controlled. Additionally, with no metrics for the magnitude of the response in each individual, for example a comparison to advanced imaging measurements such as myocardial perfusion or to a known stimulus such as adenosine, we were unable to control or account for potential differences in the breathing maneuver. A strict, standardized protocol could be implemented in a prospective trial, limiting between-site variation with direct comparisons to known stress techniques. Using a different hyperventilation technique, Alexopolous *et al*. found that the heart rate response was not as strong with hyperventilation in comparison to exercise^30^. In a small group of controls included in this analysis, we previously published that the heart rate response to hyperventilation was comparable to that of adenosine^43^. This comparison has not yet been investigated in patients.

Reproducibility analysis is not available for the patient groups, however, the test-retest reliability in 20 healthy controls suggests that HRR_HV_ is highly reproducible. Finally, HR was measured from ECG tracings inside a magnetic environment that may be subject to artifacts. The overall quality of ECG however was monitored during the scan. While the hemodynamic effects of hyperventilation are more extensively studied^30,44^, future assessments could look into the effect, reproducibility and feasibility of other breathing maneuvers such as breath-holding or paced breathing protocols.

The present study suggests that a hyperventilation-induced increase in heart rate (HRR_HV_) is attenuated in patients with cardiovascular disease. HRR_HV_ should be further investigated for its potential utility as a simple, accessible, and inexpensive pre-screening test with the potential to help improve patient selection for more expensive diagnostic modalities. A prospective study with a tightly protocolized breathing maneuver and a larger sample size should be conducted to evaluate a more accurate cut-off between healthy and disease populations while also assessing any potential adverse effects. The goal of future assessments would be to use more universal HR measuring devices that are publicly available including newer wearable devices that report additional variables such as heart rate variability.

## Methods

### Study design

The primary aim of this retrospective pooled analysis was to investigate differences in the heart rate responses to a short period of hyperventilation between controls and patients with cardiovascular disease. Data was collected from studies that were conducted between 2010 and 2018 at the Bern University Hospital in Switzerland, the Stephenson Cardiac Imaging Centre, the Montreal Heart Institute, and the McGill University Health Centre in Canada^19–21,45–47^ (NCT03050346, NCT02233634 of original studies). Hemodynamic responses to HR was not the defined end-point in any of the original studies. We pooled data from studies at these sites that applied the specific 60 s hyperventilation technique in their study in rest conditions, based on the protocol first used by Guensch *et al*.^45^. Nine studies were included with a total of 282 participants, each of which measured the heart rate response to 60 s of hyperventilation in healthy controls, patients with known or suspected CAD, or HF patients. Each of the included studies used the same 60 s hyperventilation technique in addition to other breathing maneuvers as part of a cardiac magnetic resonance (MR) imaging protocol.

### Hyperventilation procedure

Following a period of normal breathing, resting HR was recorded, after which hyperventilation commenced at a pace of at least 30 breaths per minute for 60 seconds. Immediately after, the post-hyperventilation heart rate was recorded, and HRR_HV_ was calculated as the relative difference to the resting HR. HR was recorded with an MRI-compatible ECG tracer, typically using three to four leads and directly recorded onto the images which could then be verified post-exam. Demographic information, along with ventricular function parameters were obtained on the day of the procedure.

### Participants

In all studies, healthy controls were screened for disorders and medications that are known to affect the cardiovascular and respiratory systems. Participants from the studies were combined and re-classified into five groups: healthy controls, patients who presented with CAD symptoms but had no significant coronary stenosis as evaluated via angiography (CAD−/HF− group), angiographically validated CAD patients (CAD + group), HF patients (HF + group), and patients with both HF and a history of CAD (CAD+/HF + group). Patients in the CAD + group had at least one untreated coronary stenosis ≥50% of the diameter as evaluated by angiography at the time of the hyperventilation procedure. Patients with heart failure (HF+) included patients both with reduced (<50%) and preserved (≥50) ejection fraction, had no known history of CAD and were presenting with heart failure symptoms, with either New York Heart Association classification of ≥2 or a positive B-type natriuretic peptide (BNP) or N-terminal pro B-type natriuretic peptide (NTproBNP). CAD+/HF + patients met the HF criteria and had a history of CAD, while patients in the CAD-/HF- group were patients without heart failure or a previous history of CAD who had been referred for an invasive angiography at the time of the hyperventilation procedure for the suspicion of CAD, with no relevant obstructive stenosis found on the resulting exam. Patients with implantable cardioverter defibrillators or pacemakers were excluded. More specific inclusion and exclusion criteria can be found in the relevant publications and in the NCT registry.

Each study was approved by the local research boards at the aforementioned sites, the Cantonal Ethics Committee of Bern, Bern, Switzerland, the Conjoint Health Research Ethics Board, Alberta, Canada, and the Montreal Heart Institute Research Ethics Board and McGill University Health Centre Research Ethics Board, Quebec, Canada. Written informed consent was obtained from all subjects. The data included in this specific analysis were either published with data available upon request, conducted on participants who gave consent to secondary use of their data, or were granted approval for secondary use of data by local research ethics boards. All methods were carried out in accordance with relevant guidelines and regulations.

### Data analysis

Statistical analysis and presentation were performed primarily with MATLAB (R2018a with the Image Processing Toolbox and the Statistics and Machine Learning Toolbox as well as the plotSpread function^48^) and GraphPad Prism (GraphPad Software version 8.0 for Mac OS, La Jolla California USA).

Firstly, the HRR_HV_ was compared to participant demographic variables using correlation analysis for each group. To compare the data between the individual groups, a sub-group of healthy controls 55 years and older was used as the reference. Demographic characteristics (age, body mass index (BMI), diastolic and systolic blood pressure, gender, ejection fraction, cardiac index, resting HR) were compared across groups, using ANOVA for continuous variables and a non-parametric ANOVA for categorical variables, with Bonferroni or Dunn’s multiple comparison post-hoc analysis for a direct comparison to the healthy control sub-group (≥55 years) if appropriate. An ANOVA and post-hoc analysis were then performed for comparing HRR_HV_ between all four disease groups and the sub-group of healthy controls (≥55 years). By using this older healthy control sub-group with similar demographics to the patient groups (Table 1), no covariates were added to the statistical model.

Receiver operating characteristic (ROC) curves were used to assess preliminary cut-offs between healthy and patient groups. Subsequent analysis was performed to assess the diagnostic accuracy of the cut-offs. Prior to this assessment, all cardiovascular patient were combined into a single patient group. As age is a significant factor in HRR_HV_, an additional age-stratification selection process was conducted to make two groups with an equal number of age matched patients following previously reported procedures^49^. The participants were stratified into three age bins (40–49, 50–59, 60–72), with an equal number of patients and controls pseudo-randomized for inclusion into each bin. With this sub-analysis population, n = 54 were available for each group.

Finally, reproducibility of HRR_HV_ was also assessed in the 20 healthy controls who completed the breathing maneuver multiple times. Test-retest reproducibility was assessed with both the Lin concordance coefficient and the absolute single measure intraclass correlation coefficient (ICC) using a two-way mixed model. Unless otherwise indicated, all stats are reported as mean ± standard deviation.

## Supplementary information

Supplemental Information

Dataset 1

## Data Availability

All data generated or analyzed during this study are included in this published article (and its Supplementary Information files, Supplementary Dataset 1).
