# Supplementary material for: Hyperventilation-induced heart rate response as a potential marker for cardiovascular disease
Source: Sci Rep. 2019 Nov 29;9:17887. doi: 10.1038/s41598-019-54375-9 (PMC6884614; doi:10.1038/s41598-019-54375-9)
Supplement: Supplementary file 1 — Supplemental Information [file 41598_2019_54375_MOESM1_ESM.pdf]

SUPPLEMENTAL INFORMATION

Hyperventilation-induced heart rate response as a potential marker for cardiovascular disease

Selwynne Hawkins, BSc[1]; Dominik P Guensch, MD[2,3]; Matthias G Friedrich, MD[4,5,6] Giulia Vinco, MD[4,7]; MD; Gobinath Nadeshalingham, MSc[8]; Michel White, MD[8]; Francois-Pierre Mongeon, MD[8]; Elizabeth Hillier, BSc[1]; Tiago Teixeira, MD[8,10]; Jacqueline A Flewitt, MSc[9]; Balthasar Eberle, MD[2]; Kady Fischer, PhD[2,3]*

Supplemental Figure 1: Correlation of HRR_{HV} with Demographic Variables 2

Supplemental Figure 2: ROC Curve 3

Supplemental Figure 1: Correlation of HRR_{HV} with Demographic Variables

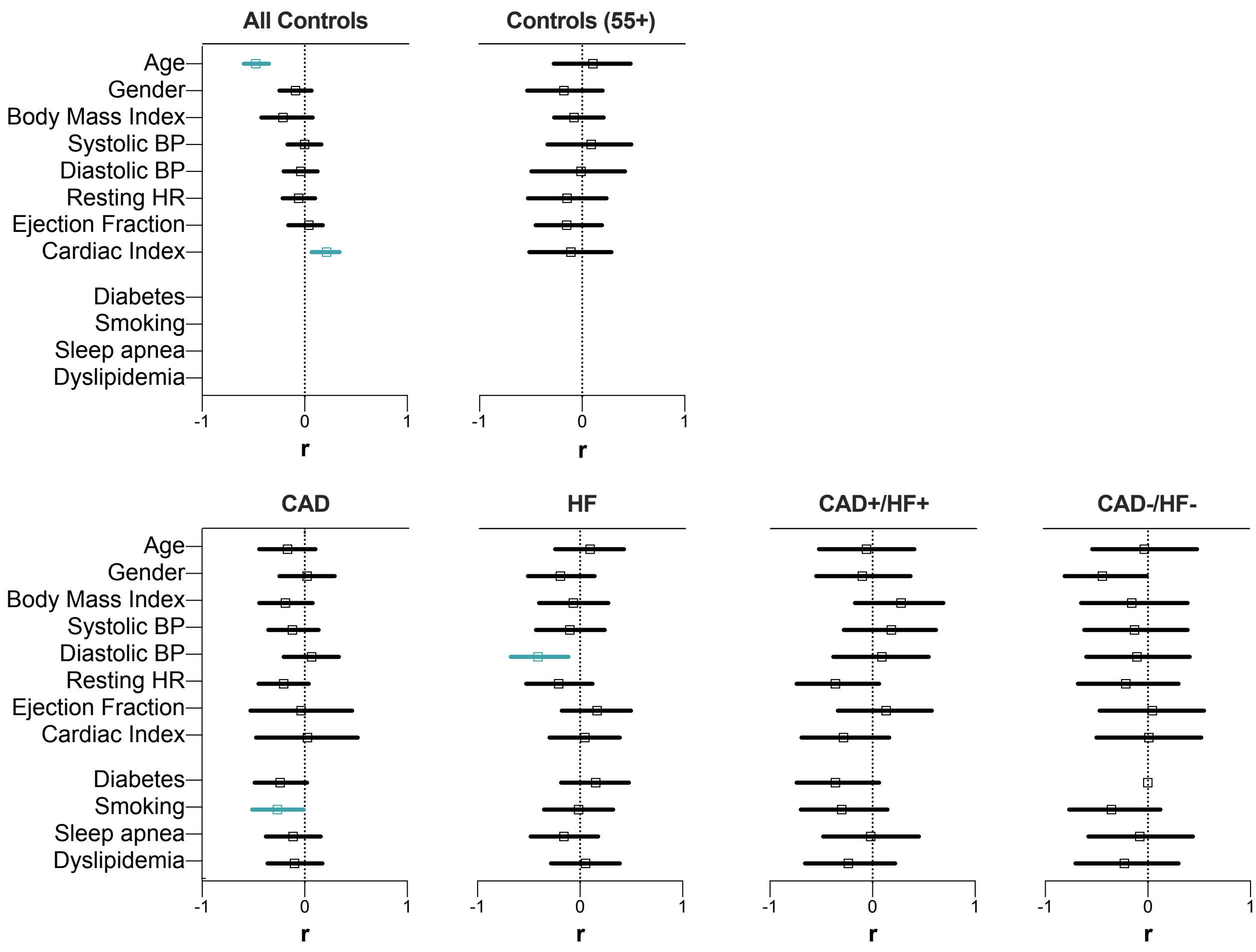

Linear analysis (*r*-coefficient with 95% confidence intervals) indicated that age, and cardiac index were associated with the heart rate response to hyperventilation (HRR_{HV}) in all controls, but were not related to the HRR_{HV} in the individual patient groups or controls aged 55 years and older. Blue markers indicate a significant correlation (*p<0.05).

Supplemental Figure 2: ROC Curve

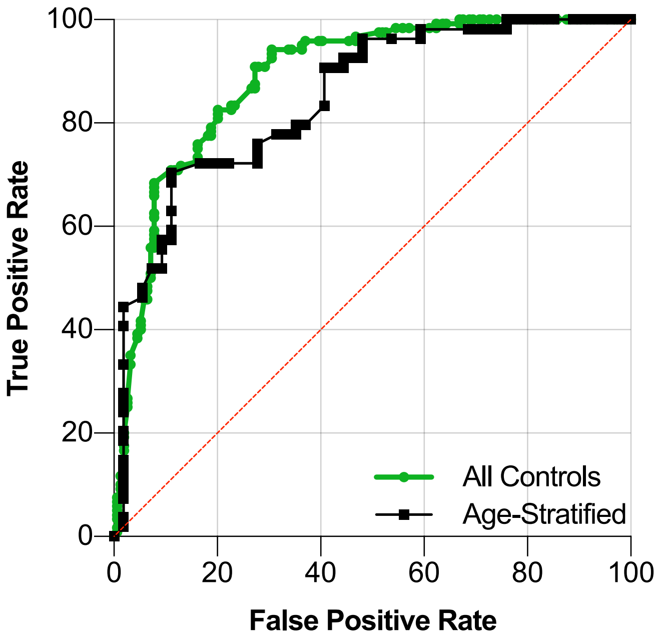

Receiver operator curve illustrating the diagnostic ability of binary classification (controls or combined cardiovascular disease group) based on the heart rate response to hyperventilation (HRR_{HV}). The green curve represents analysis of all healthy controls (n=161) in comparison to all patients (n=121), which does not account for any age differences. After age stratification (black), the difference in heart rate response remained significant (n=54 per group).
